# Tumor suppressor REIC/DKK-3 and co-chaperone SGTA: Their interaction and roles in the androgen sensitivity

**DOI:** 10.18632/oncotarget.6488

**Published:** 2015-12-02

**Authors:** Kazuhiko Ochiai, Masami Morimatsu, Yuiko Kato, Toshina Ishiguro-Oonuma, Chihiro Udagawa, Oumaporn Rungsuriyawiboon, Daigo Azakami, Masaki Michishita, Yuichi Ariyoshi, Hideo Ueki, Yasutomo Nasu, Hiromi Kumon, Masami Watanabe, Toshinori Omi

**Affiliations:** ^1^ Department of Veterinary Nursing and Technology, School of Veterinary Science, Nippon Veterinary and Life Science University, Tokyo 180-8602, Japan; ^2^ Laboratory of Laboratory Animal Science and Medicine, Department of Disease Control, Graduate School of Veterinary Medicine, Hokkaido University, Sapporo 060-0818, Japan; ^3^ Department of Biological Resources, Integrated Center for Science, Ehime University, Ehime 791-0295, Japan; ^4^ Department of Veterinary Technology Faculty of Veterinary Technology, Kasetsart University, Bangkok 10900, Thailand; ^5^ Department of Veterinary Pathology, School of Veterinary Science, Nippon Veterinary and Life Science University, Tokyo 180-8602, Japan; ^6^ Department of Urology, Graduate School of Medicine, Dentistry and Pharmaceutical Sciences, Okayama University, Okayama 700-8558, Japan

**Keywords:** REIC/DKK-3, SGTA, androgen, TCTEX-1, prostate cancer.

## Abstract

REIC/DKK-3 is a tumor suppressor, however, its intracellular physiological functions and interacting molecules have not been fully clarified. Using yeast two-hybrid screening, we found that small glutamine-rich tetratricopeptide repeat-containing protein α (SGTA), known as a negative modulator of cytoplasmic androgen receptor (AR) signaling, is a novel interacting partner of REIC/DKK-3. Mammalian two-hybrid and pull-down assay results indicated that the SGTA-REIC/DKK-3 interaction involved the N-terminal regions of both REIC/DKK-3 and SGTA and that REIC/DKK-3 interfered with the dimerization of SGTA, which is a component of the AR complex and a suppressor of dynein motor-dependent AR transport and signaling. A reporter assay in human prostate cancer cells that displayed suppressed AR signaling by SGTA showed recovery of AR signaling by REIC/DKK-3 expression. Considering these results and our previous data that REIC/DKK-3 interacts with the dynein light chain TCTEX-1, we propose that the REIC/DKK-3 protein interferes with SGTA dimerization, promotes dynein-dependent AR transport and then upregulates AR signaling.

## INTRODUCTION

Reduced expression in immortalized cells (REIC), which was discovered initially as a tumor suppressor gene, is identical to the Dickkopf-3 (DKK-3) gene, which is a member of the Dickkopf gene family [1]. REIC/DKK-3 is expressed ubiquitously in normal cells, whereas its expression is downregulated significantly in various types of cancer cells, including prostate cancer [2–4]. We and other investigators have demonstrated that REIC/DKK-3 overexpression by adenoviral or plasmid vectors induces apoptosis in multiple cancer cell lines, but not in normal cells, via c-Jun-NH2-terminal kinase (JNK) and c-Jun activation [5–10]. Our previous study also indicated that REIC/DKK-3 overexpression by adenoviral vectors triggers apoptosis induction via endoplasmic reticulum (ER) stress signaling [11,12]. Various studies have shown that REIC/DKK-3 downregulation is closely associated with the pathological malignancy of clinical specimens of various cancer types [2,13,14]. Moreover, REIC/DKK-3 seems to have distinct anti-oncogenic functions via a mechanism involving the cell cycle and anti-apoptotic processes. Although the expression levels of the REIC/DKK-3 protein itself or the specific processes involved in ER stress signaling could be important for the anti-oncogenic functions of REIC/DKK-3 [8], the molecules that interact with the REIC/DKK-3 protein and how this protein prevents or suppresses malignant transformation have not yet been elucidated.

Cytoplasmic dynein is a microtubule-based molecular motor that plays an essential role in the locomotion of various intracellular proteins and cell organelles. The dynein motor complex consists of multiple proteins, including two heavy chains (HC, ∼530 kDa), two intermediate chains (IC, ∼74 kDa), four light intermediate chains (∼60 kDa) and several light chains (8, 14 and 22 kDa) [15]. We demonstrated previously that TCTEX-1 [16], a 14 kDa light chain of the cytoplasmic dynein motor complex that plays an important role as an adaptor protein between the dynein motor and various proteins, directly interacts with REIC/DKK-3 [17–21]. However, the significance and cellular consequences of this interaction have been poorly defined, and the molecular mechanisms underlying how REIC/DKK-3 is implicated in dynein motor dynamics remain largely elusive.

Small glutamine-rich tetratricopeptide repeat-containing protein α (SGTA) is a co-chaperone that is known to interact with heat shock protein 70 (HSP70) and HSP90 and with the steroid receptor complex, including the androgen receptor (AR) [22–25]. SGTA is expressed ubiquitously in normal mammalian tissues and plays critical roles in a variety of biological processes, including cell division and differentiation, as well as viral infection [26–29]. SGTA can substantially influence the actions of androgens and is consequently involved in hormone-mediated carcinogenesis [23,24,29]. During the past decade, the intracellular roles of SGTA have been intensively examined. The dimerized form of SGTA was reported to be a negative regulator of AR transport to the nucleus by inhibiting the link between the cytoplasmic AR complex and the dynein motor complex [23,24,29–32]. Although SGTA is a negative regulator of AR signaling and most likely inhibits dynein motor-dependent AR transport, little is known regarding the association between the AR complex, SGTA and the dynein motor complex. In addition, the interaction between REIC/DKK-3 and TCTEX-1 may be involved in the dynein motor-dependent transport of various molecules, including the AR, although whether this interaction affects SGTA-inhibited AR transport and signaling remains unknown.

We explored the cytoplasmic binding partners of the REIC/DKK-3 protein; this study demonstrates that the SGTA protein is a novel REIC/DKK-3 interaction partner. Because the dimerized form of SGTA is a component of the AR complex and is known to be a negative modulator of cytoplasmic AR signaling, we investigated the ability of REIC/DKK-3 to modify androgen signaling in human prostate cells. In this manuscript, we describe the roles of cytoplasmic REIC/DKK-3 in SGTA-inhibited AR signaling and its possible function in the androgen sensitivity.

## RESULTS

### SGTA is a novel REIC/DKK-3-interacting protein

**Figure 1:**
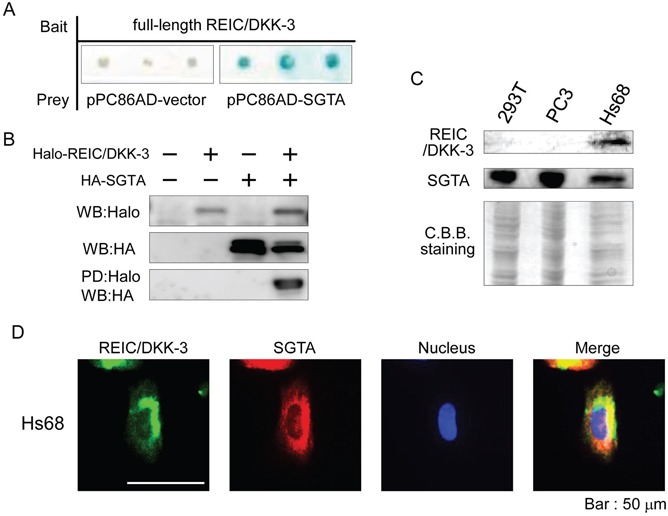
The interaction between the REIC/DKK-3 and SGTA proteins **A.** The yeast two-hybrid analysis was conducted using pPC86 (AD)/full-length human SGTA (derived from a normal heart cDNA library) and pDBLeu (BD)/full-length human REIC/DKK-3 plasmids. The blue colonies indicate those with an interaction between the two proteins. **B.** For the pull-down (PD) assay, the full-length cDNA of human REIC/DKK-3 and SGTA was cloned into the pFN21A and pMACS Kk.HA-C plasmids, respectively. Cell lysates from Halo-tagged REIC/DKK-3- and/or HA-tagged SGTA-transfected 293T cells were analyzed. The sample pulled down using Halo-tagged REIC/DKK-3 was analyzed by Western blotting (WB) using anti-HA antibody. **C.** REIC/DKK-3 and SGTA protein expression in 293T, PC3 and Hs68 cells was analyzed by Western blotting. Coomassie Brilliant Blue (CBB) staining of the membrane is shown as a loading control. **D.** The co-localization of REIC/DKK-3 and SGTA was examined by double immunofluorescence staining and observed by fluorescence microscopy. The images in green and red show the intracellular localization of REIC/DKK-3 and SGTA, respectively. The areas of overlap between REIC/DKK-3 and SGTA are shown in yellow in the merged image.

To identify the novel interacting partners for the REIC/DKK-3 protein, a yeast two-hybrid screen was conducted using cDNA libraries derived from a normal human heart and prostate and from a prostate adenocarcinoma. We added the cDNA library from a normal heart because higher REIC/DKK-3 mRNA and protein levels in the heart tissue compared to other tissues were previously reported [1,3]. Among the cDNA clones that reproducibly activated the reporters, some candidates were identified as independent. The positive clones were validated by plasmid rescue, retransformation and growth on reporter-selective plates. As a result, from the normal heart and prostate adenocarcinoma cDNA libraries, SGTA was found to be a novel protein that interacts with full-length human REIC/DKK-3 (Figure 1A, normal heart result).

To confirm the interaction between REIC/DKK-3 and SGTA, we performed an *in vitro* pull-down (PD) assay in 293T cells. The lysates from the cells expressing Halo-REIC/DKK-3 and HA-SGTA fusion proteins were analyzed for binding between REIC/DKK-3 and SGTA. Definite SGTA binding with REIC/DKK-3 was detected in the samples pulled down by Halo in the cell lysates as confirmed by Western blot (WB) analysis with anti-HA antibody (Figure 1B). Therefore, the interaction between REIC/DKK-3 and SGTA was demonstrated by both yeast two-hybrid and mammalian PD assay systems. The expression of both REIC/DKK-3 and SGTA proteins was observed in human Hs68 fibroblasts; however, only SGTA expression was detected in 293T cells and in PC3 human prostate cancer cells (Figure 1C).

To further verify the interaction between REIC/DKK-3 and SGTA in mammalian cells, immunocytochemistry was performed in Hs68 fibroblasts using anti-REIC/DKK-3 and anti-SGTA antibodies. Immunostaining revealed that REIC/DKK-3 and SGTA showed cytoplasmic localization with a similar distribution pattern, suggesting that their interaction and functions occur in the same locations (Figure 1D).

### The REIC/DKK-3 and SGTA interaction involves the N-terminal regions of both proteins

**Figure 2:**
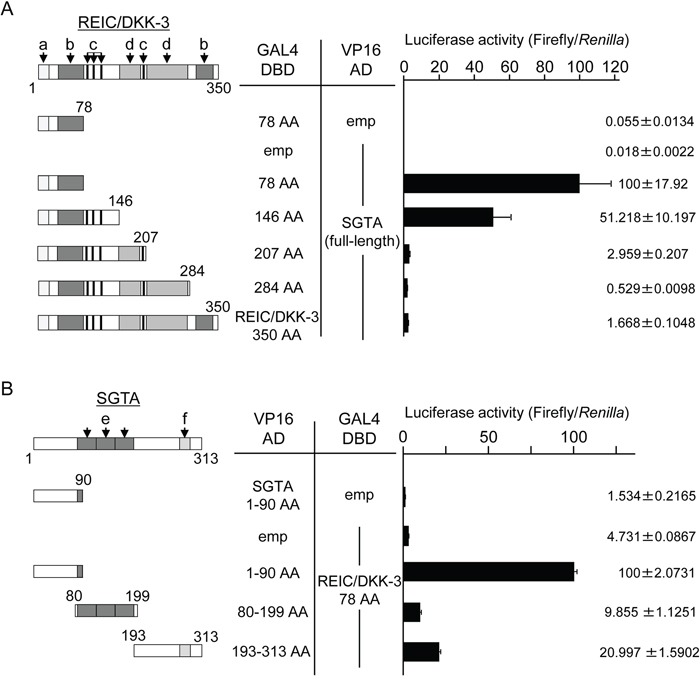
The determination of the binding region between REIC/DKK-3 and SGTA **A.** The interactions of various lengths of REIC/DKK-3 and full-length SGTA were examined in a mammalian two-hybrid assay. The left panel depicts the various lengths of the REIC/DKK-3 fragments examined. The numbers correspond to the AAs in REIC/DKK-3. Two-hybrid constructs of each REIC/DKK-3 fragment (GAL4 DBD) and full-length SGTA (VP16 AD) were co-transfected into 293T cells. Luciferase activity was measured to determine the binding activities with SGTA; the results are shown in the right panel. The regions of REIC/DKK-3 were as follows: a, signal peptide; b, coiled-coil structure; c, N-glycosylation site; and d, cysteine-rich domain. The results were obtained from three independent experiments. The bar graphs show the luciferase activities on the interaction of REIC/DKK-3 78 AA-GAL4 DBD and full SGTA-VP16 AD column as 100%. **B.** The interactions of various lengths of SGTA (VP16 AD) with the 78 AA fragment of REIC/DKK-3 (GAL4 DBD) were examined in a mammalian two-hybrid assay. The left panel presents the 1-90 AA, 80-199 AA and 193-313 AA regions of SGTA. The binding activities of these fragments with the REIC/DKK-3 domain are shown in the right panel. The regions of SGTA were as follows: e, TPR domain, and f, glutamine-rich domain. The results are derived from three independent experiments. The bar graphs show the luciferase activities on the interaction of SGTA 90 AA-VP16 AD and REIC/DKK-3 78 AA-GAL4 DBD column as 100%.

Next, we attempted to determine the binding regions of the REIC/DKK-3 and SGTA proteins that are involved in their interaction by performing a mammalian two-hybrid assay. By co-transfecting different lengths of REIC/DKK-3 and SGTA in the GAL4 DBD and VP16 AD plasmids, respectively, the luciferase activity of the 293T cell lysates was altered based on whether an interaction between REIC/DKK-3 and SGTA occurred. The most significant binding with SGTA was observed for the N-terminal REIC/DKK-3 fragment (1–78 amino acids: AA) (Figure 2A). The N-terminal region (1–90 AA) of SGTA strongly interacted with these 78 AAs of REIC/DKK-3 (Figure 2B).

### REIC/DKK-3 competitively interferes with SGTA-SGTA dimerization

**Figure 3:**
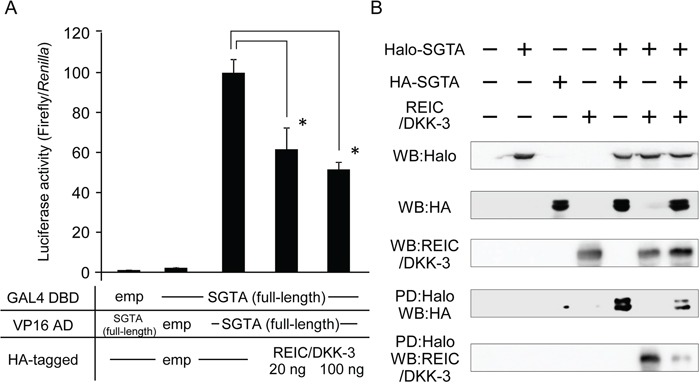
The interference of REIC/DKK-3 with SGTA-SGTA dimerization **A.** The interference of REIC/DKK-3 with SGTASGTA dimerization was analyzed in a mammalian two-hybrid assay. Two-hybrid constructs of full-length SGTA (GAL4 DBD) and fulllength SGTA (VP16 AD) were co-transfected into 293T cells. HA-tagged full-length REIC/DKK-3 was also transfected under the indicated conditions. Luciferase activity was measured to determine the extent of SGTA-SGTA dimerization; the results are shown in the bar graphs. The data are from four independent experiments. The bar graphs show the luciferase activities on the interaction of SGTA-DBD, SGTA-AD and HA-tagged empty vector column as 100%. *, A significant difference was observed. B. For the pull-down (PD) assay, the full-length human SGTA cDNA was cloned into both the pFN21A and pMACS Kk.HA-C plasmid vectors. A pDNR-CMV vector encoding the fulllength REIC/DKK-3 was also used. These plasmids were co-transfected, and the cell lysates from Halo-tagged SGTA- and/or HA-tagged SGTA- and/or REIC/DKK-3-transfected 293T cells were analyzed. The samples pulled down by Halo-tagged SGTA were analyzed by Western blotting (WB) with the indicated antibodies.

To elucidate whether the REIC/DKK-3-SGTA interaction affects SGTA-SGTA dimerization, the extent of SGTA-SGTA dimerization after REIC/DKK-3 expression was measured using a mammalian two-hybrid assay. Two-hybrid constructs of full-length SGTA (GAL4 DBD) and full-length SGTA (VP16 AD) were co-transfected into 293T cells, and the full-length REIC/DKK-3 construct was transfected to determine whether it interfered with SGTA dimerization. The luciferase activity was significantly downregulated in a REIC/DKK-3 dose-dependent manner, indicating that intracellular REIC/DKK-3 inhibited SGTA-SGTA dimerization, and the strength of interference was approximately 50% (Figure 3A).

To further verify the interference of SGTA-SGTA dimerization by REIC/DKK-3 expression, a PD assay was performed in 293T cells. Halo-tagged and HA-tagged full-length human SGTA proteins were co-expressed with the full-length REIC/DKK-3 protein as the interfering factor, and the cell lysates were analyzed. In the samples pulled down by Halo-tagged SGTA, clear bands for HA-tagged SGTA and REIC/DKK-3 were detected by WB analysis and demonstrated the binding of SGTA (Halo-tagged) and SGTA (HA-tagged) (for dimerization) and the interaction between REIC/DKK-3-SGTA (Halo-tagged) (Figure 3B). Significantly reduced densities of these bands were noted for the triple treatment with the expression plasmids, demonstrating competition between the REIC/DKK-3 protein and non-dimerized SGTA (HA-tagged) during SGTA-SGTA dimer formation.

### SGTA downregulates AR signaling, and full-length REIC/DKK-3 recovers this downregulation

**Figure 4:**
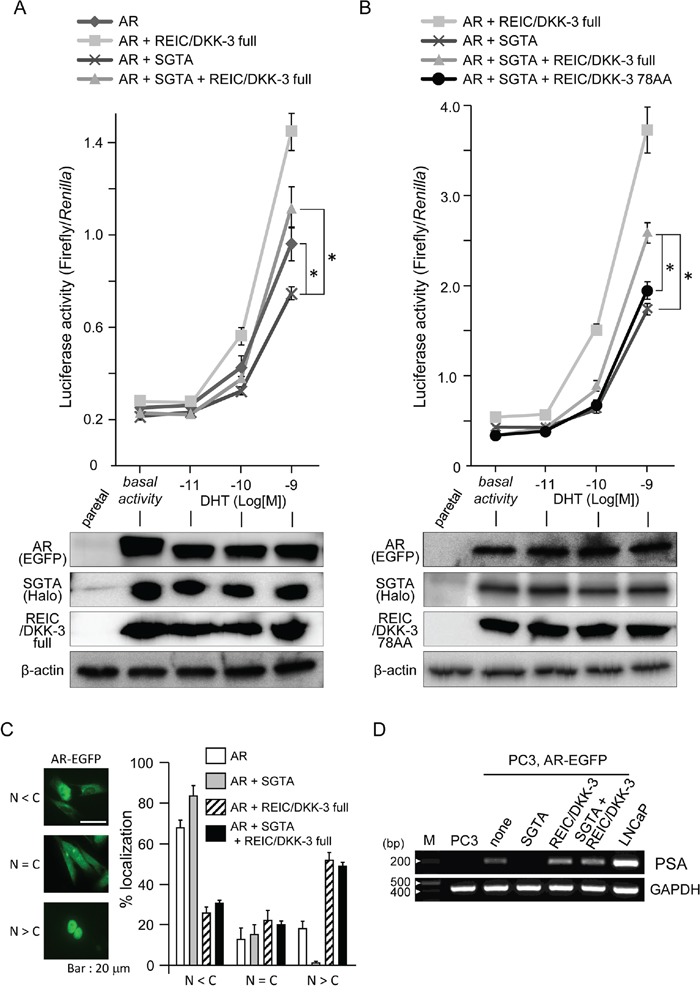
The modification of AR sensitivity, AR localization and PSA expression by SGTA and/or REIC/DKK-3 expression in pEGFP-C1-AR-transfected PC3 cells **A.** The effects of full-length SGTA and full-length REIC/DKK-3 expression on AR activity were examined in PC3 human prostate cancer cells. Under co-transfection with AR, SGTA and/or REIC/DKK-3 plasmids, the cells were transfected with the p159-pPr-Luc plasmid vector and were treated with DHT. The value for the baseline was obtained using the vehicle control. The results are derived from three independent experiments. *, A significant difference was observed. Western blotting analyses of parallel samples were depicted under of the graphs. **B.** The effects of the expression of full-length REIC/DKK-3 and its 78 AA fragment on AR activity were compared in PC3 cells under co-expression with full-length SGTA. The results are derived from three independent experiments. *, A significant difference was observed. Western blotting analyses of parallel samples were depicted under of the graphs. **C.** Localization of AR-EGFP after DHT (1 nM) treatment was analyzed in AR-transfected PC3 cells. The left panel shows the representative localization of AR-EGFP for the three categories (nucleus: N < cytoplasm: C, N = C, N > C) examined by fluorescence microscopy. The green fluorescence in the images shows the intracellular localization of AR-EGFP, and AR localization was monitored based on GFP fluorescence. The right panel shows the results of the analyses of AR nuclear translocation after the treatments. Images containing at least 100 cells were evaluated in each treatment, and the cellular category for AR localization was determined. The results are from three independent experiments. **D.** Semiquantitative RT-PCR was performed in parental PC3 and LNCaP cells and in pEGFP-C1-AR-transfected PC3 cells under the indicated plasmid co-transfections. The cells were incubated with 1 nM DHT for 5 days, and the mRNA level of endogenous PSA was examined by RT-PCR. GAPDH mRNA expression was used as the internal control.

To examine the roles of intracellular SGTA and REIC/DKK-3 and their interaction in AR signal transduction, we expressed and co-expressed these proteins in PC3 prostate cancer cells under additional AR co-expression. PC3 cells lack a functional AR [33], and dihydrotestosterone (DHT)-dependent AR signaling activation was observed in these cells after additional AR expression [33]. The extent of AR signaling activation was measured by a probasin promoter-driven luciferase reporter assay. This AR-responsive promoter assay showed that SGTA significantly suppressed androgen signaling (Figure 4A). We also demonstrated that AR signaling activation in cells co-expressing SGTA was observed when these cells were also transfected with the full-length REIC/DKK-3 (1–350 AA) construct but not when transfected with the partial REIC/DKK-3 (1–78 AA) construct (Figures 4A, 4B). The results of the AR-EGFP localization ratio in the cytoplasm (C) and nucleus (N) are presented according to the 3 indicated categories (Figure 4C). AR-EGFP was predominantly located in the cytoplasm when SGTA was expressed. The expression of full-length REIC/DKK-3 facilitated AR nuclear translocation with and without SGTA expression.

To further investigate whether SGTA and/or REIC/DKK-3 expression modifies the AR-dependent endogenous transcriptional system in AR-EGFP-transfected PC3 cells, we evaluated the changes in endogenous prostate-specific antigen (PSA) transcription at the mRNA level. The cells were incubated with 1 nM DHT for 5 days, and then mRNA samples were obtained for semiquantitative RT-PCR analysis. PSA mRNA transcription was not detected in the parental PC3 cells, while the parental LNCaP cells strongly expressed PSA mRNA (Figure 4D).

### A lack of the putative SGTA binding domain of REIC/DKK-3 abolished the interference of SGTA dimerization

**Figure 5:**
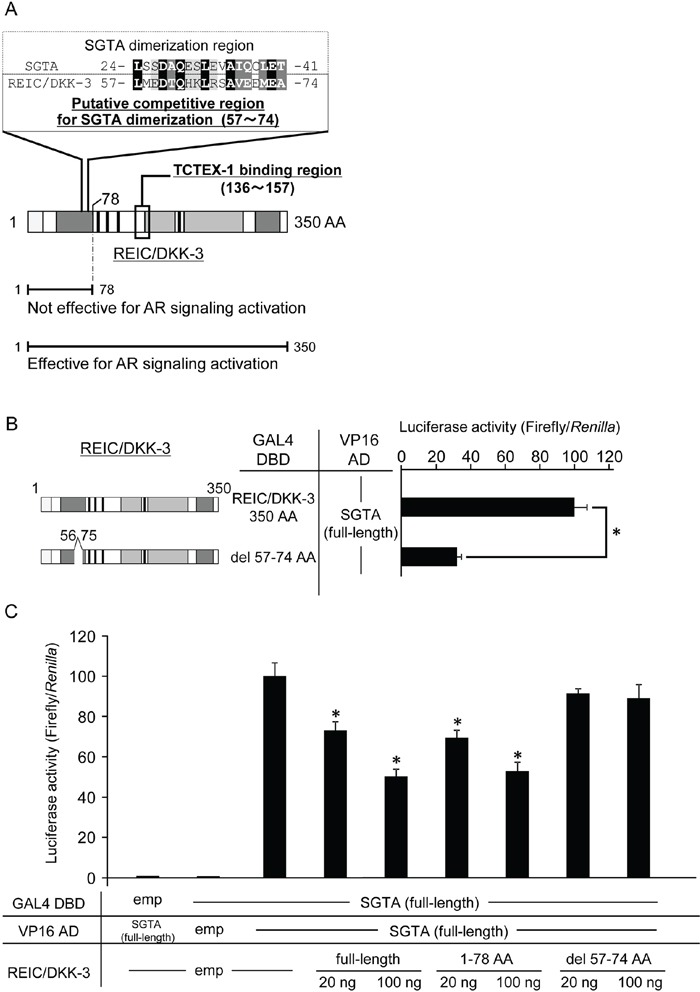
The proposed binding region of the REIC/DKK-3-SGTA interaction **A.** Based on the results from Figure 2, sequence homology analysis was performed between the 1–78 AA region of REIC/DKK-3 (SGTA binding region) and the reported SGTA dimerization region (1–80 AA) [24,30,31]. The analysis revealed the putative competitive region (57–74 AA) of REIC/DKK-3 that affects SGTA dimerization. Sequence alignment analysis between the competitive region of REIC/DKK-3 and the affected part (24–41 AA) of the SGTA dimerization region was performed using the ClustalW software program (http://www.genome.jp/tools/clustalw/). The completely conserved residue is shaded in black, and the residues belonging to strongly similar groups are shaded in gray. The residues exhibiting weaker similarity are shaded in light gray. **B.** The interactions of REIC/DKK-3, which was deleted the putative binding region (57–74 AA), with SGTA and full-length SGTA was examined in a mammalian two-hybrid assay. The left panel depicts the REIC/DKK-3 fragment examined, and the results are shown in the right panel. The data are from three independent experiments. The bar graphs show the luciferase activities on the top column as 100%. *, A significant difference was observed. **C.** The interference activity of REIC/DKK-3 (del 57–74) with the SGTA-SGTA dimerization was analyzed in a mammalian two-hybrid assay. The data are from three independent experiments. The bar graphs show the luciferase activities on the interaction of SGTA-DBD, SGTA-VP16 AD and HA-tagged empty vector column as 100%. *, A significant difference was observed.

Next, we attempted to elucidate a putative competitive region of REIC/DKK-3 for SGTA dimerization using the ClustalW program, and we found a highly conserved region between REIC/DKK-3 (57–74 AA) and SGTA (24–41 AA) (Figure 5A). To test the hypothesis that these two proteins bind, we performed a mammalian two-hybrid assay between a deletion construct of REIC/DKK-3 in which 57–74 AA were deleted (del 57–74) and full-length SGTA. Luciferase activity was significantly reduced compared to that for the binding of full-length REIC/DKK-3 and SGTA (Figure 5B). Furthermore, we analyzed the interference activities of the deletion region del 57–74 for SGTA dimerization. Compared with the full-length and 78 AA REIC/DKK-3 constructs, del 57–74 did not interfere with SGTA dimerization (Figure 5C).

### TCTEX-1, a binding partner of REIC/DKK-3, positively enhances REIC/DKK-3-induced AR signaling

**Figure 6:**
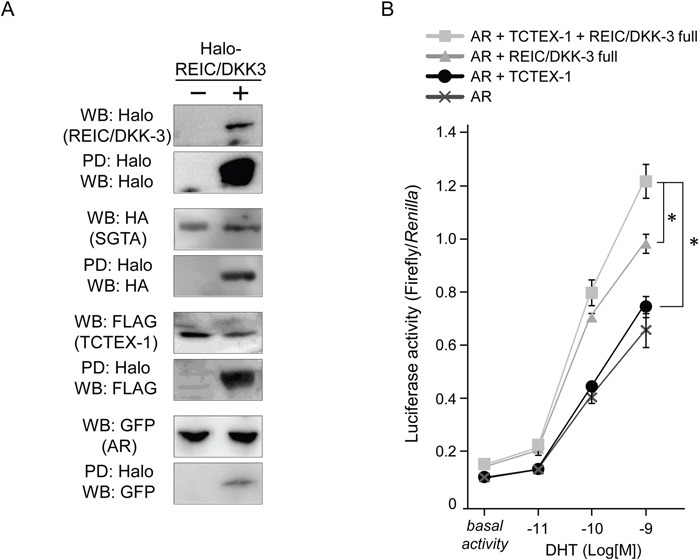
REIC/DKK-3 is a part of the AR complex including SGTA and TCTEX-1 **A.** The pull-down (PD) assays of AR complex. Halo-tagged REIC/DKK-3, HA-tagged SGTA, FLAG-tagged TCTEX-1 and GFP-tagged AR were expressed in PC3 cell. Halo tag pull-down was performed. For the PD assay of the investigation of the component including AR complexes, Halo-tagged REIC/DKK-3, HA-tagged SGTA, FLAG-tagged TCTEX-1 and GFP-tagged AR were expressed in PC3 cells. Cell lysates from both plasmids transfected PC3 cells were analyzed. The sample pulled down by Halo-tagged REIC/DKK-3 was analyzed by Western blotting (WB) using anti-Halo, -HA, -FLAG and -GFP and antibodies **B.** The effects of REIC/DKK-3 and TCTEX-1 expression on AR activity were examined in PC3 cells. Under co-transfection with AR, REIC/DKK-3 and/or TCTEX-1 plasmids, the cells were transfected with the p159-pPr-Luc plasmid vector and were treated with DHT. The value for the baseline was obtained using the vehicle control. The results are derived from three independent experiments. *, A significant difference was observed.

To investigate the component of AR-REIC/DKK-3 complex, we tried to pull-down AR, SGTA and TCTEX-1 by Halo-tagged REIC/DKK-3. The lysates from PC3 cells expressing Halo-REIC/DKK-3, GFP-AR, FLAG-TCTEX-1 and HA-SGTA fusion protein were analyzed for the binding between REIC/DKK-3 and the components of AR complex. The binding between REIC/DKK-3 with other proteins was detected by a WB analysis using an anti-tag protein antibodies. Definite REIC/DKK-3 binding with the SGTA, TCTEX-1 and AR were disclosed in the Halo-PD samples from the cell lysates (Figure 6A).

To examine the functional effects of TCTEX-1 on the AR signal transduction with REIC/DKK-3, we expressed and co-expressed REIC/DKK-3 and/or TCTEX-1 proteins in PC3 under additional AR co-expression. This AR-responsive promoter assay showed that REIC/DKK-3 expressed alone, and REIC/DKK-3 and TCTEX-1 co-expressed samples significantly extended androgen signaling (Figure 6B). These results indicate that the TCTEX-1 positively correlates with the enhancement of the AR signal transduction induced by REIC/DKK-3 expression with DHT treatment. Thus, significant involvement of the interaction between REIC/DKK-3 and TCTEX-1, a light chain of the dynein motor complex, occurs during AR transduction, suggesting that these proteins functionally interact during the AR maturation process.

## DISCUSSION

Here, we demonstrated that SGTA, which is known to bind to AR and HSP70/HSP90 proteins [22–24], is a novel REIC/DKK-3-binding protein. The results indicate that the interaction between REIC/DKK-3 and SGTA involves the N-terminal regions of both proteins. As for the region of REIC/DKK-3, it is likely that the other regions than the N-terminal 78 AA region could physically cover the N-terminal 78 AA region and decrease the binding activity with SGTA (Figure 2A). We showed that cytoplasmic REIC/DKK-3 interferes with the dimerization of SGTA and abolishes the function of SGTA as a negative regulator of the AR, resulting in enhanced AR sensitivity in human prostate cancer cells. Thus, we showed that REIC/DKK-3 plays a role in the upregulation of AR signaling by interacting with SGTA in human prostate cells.

Androgen sensitivity is regulated by the transport system of the AR complex, which consists of HSP70/HSP90 chaperones and the co-chaperone and TPR (tetratricopeptide repeat)-motif containing protein SGTA [23,24,29]. Through the TPR-motif, the dimerized SGTA molecules interact with AR, and then chaperone and maintain the AR complex in an androgen-responsive, but inactive, state [23,29]. After ligands bind to the AR, the dimerized SGTA is thought to be released from the AR complex, and then AR moves to the nucleus along the dynein motor axis [23,24,29]. Our experiments demonstrated that REIC/DKK-3 interacts with SGTA and disturbs SGTA dimerization. We also demonstrated that the REIC/DKK-3 protein increases AR activity when SGTA is co-expressed, indicating that REIC/DKK-3 activates AR transport by binding to and interfering with the negative regulator SGTA. Based on the results shown in Figure 2, a sequence homology analysis between the 1–78 AA region of REIC/DKK-3 (SGTA-binding region) and the reported SGTA dimerization region (1–80 AA) [24,30,31] was performed to determine the possible competitive region of REIC/DKK-3 for inhibiting SGTA dimerization. The homology analysis revealed a putative competitive region (57–74 AA) of REIC/DKK-3 for inhibiting SGTA dimerization (Figure 5A). The sequence alignment analysis using the ClustalW program demonstrated significantly conserved or similar residues between the competitive region of REIC/DKK-3 and a portion (24–41 AA) of the SGTA dimerization region. We finally demonstrated that deleting the putative SGTA-binding domain (57–74 AA) of REIC/DKK-3 abolished its ability to interfere with SGTA dimerization, suggesting that the REIC/DKK-3 domain is essential for the upregulation of AR signaling by interacting with SGTA. Intensive analyses focusing on the SGTA subdomains were conducted previously and indicated that the SGTA region (21–40 AA) is necessary for the effects of SGTA on the activity of both exogenous and endogenous AR [30]. The previous study also analyzed the sequence conservation of the SGTA dimerization region from nine different species, including humans, and revealed that the SGTA region (27–41 AA) exhibited 100% sequence identity across species, showing an evolutionarily highly conserved region [30]. Notably, these regions that are essential for the functions of SGTA are nearly identical to the currently proposed SGTA region (24–41 AA) that is required for REIC/DKK-3 binding.

**Figure 7:**
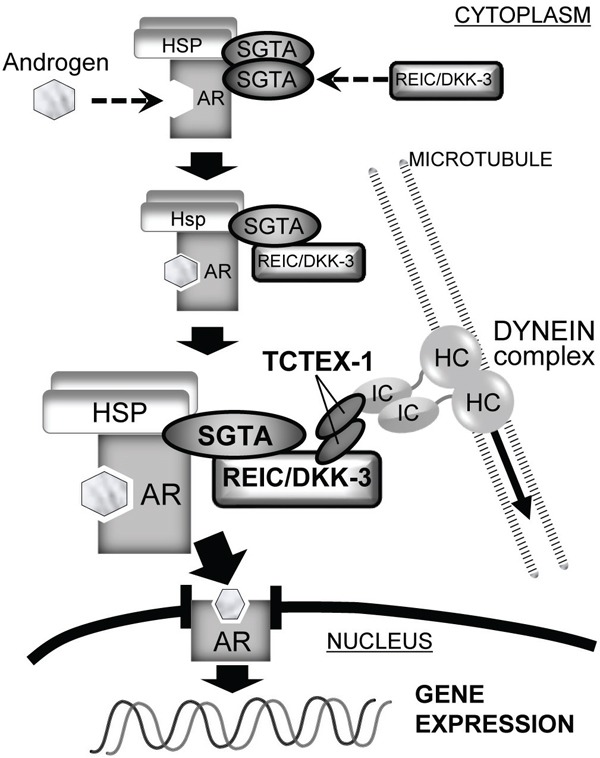
The role of the REIC/DKK-3-SGTA interaction in AR signaling The proposed role of the REIC/DKK-3-SGTA interaction for AR signaling is shown. In the schematic overview, AR signaling consists of AR maturation and AR transport to the nucleus by the dynein motor axis. SGTA dimerization acts to stabilize the AR complex and limits AR transport to the nucleus. When androgen binds to the AR and REIC/DKK-3 interacts with SGTA, abrogating SGTA dimerization, the AR complex is activated, and dynein motor-dependent AR transport (signaling) is facilitated. In this model of AR transport by the dynein motor complex, the interaction of REIC/DKK-3 with TCTEX-1 is also necessary. Thus, the intracellular interactions of REIC/DKK-3-SGTA and REIC/DKK-3-TCTEX-1 are both involved in AR maturation and signaling.

In addition, we previously reported the interaction of REIC/DKK-3 with TCTEX-1, a 14 kDa light chain of the cytoplasmic dynein motor complex, and determined the TCTEX-1-binding region (136–157 AA) of REIC/DKK-3 [16]. The current results shown in Figure 4 indicate that AR signaling activation when SGTA was co-expressed was observed in the cells expressing full-length REIC/DKK-3 (1–350 AA) but not in those expressing the REIC/DKK-3 fragment (1–78 AA). Both the SGTA-binding region and TCTEX-1-binding region of REIC/DKK-3 are presumably required for AR transport and for androgen signaling activation. Furthermore, REIC/DKK-3 expression led to a full recovery of the downregulation of AR sensitivity caused by SGTA expression (Figure 4A), indicating that the interaction of REIC/DKK-3 with SGTA is crucial for abolishing the suppressive role of SGTA in dynein motor-dependent AR transport. Additionally, our hypothesis that REIC/DKK-3 promotes AR transport and upregulates AR signaling by abolishing the suppressive effects of SGTA is further strengthened by the results obtained in the AR nuclear translocation assay (Figure 4C) and quantitative analysis of endogenous PSA transcription (Figure 4D). As for the interaction of REIC/DKK-3 with TCTEX-1, additional TCTEX-1 expression led to a significant upregulation of AR sensitivity in comparison to the case with REIC/DKK-3 expression only (Figure 6B), indicating that the interaction between REIC/DKK-3 and TCTEX-1 has a positive role in dynein motor-dependent AR signaling. The pull-down assays of AR complex demonstrated that the complex composed of AR, SGTA, REIC/DKK-3 and TCTEX-1 (Figure 6A). Taken together, based on the significant interaction and co-localization of REIC/DKK-3 and SGTA and of REIC/DKK-3 and TCTEX-1, a light chain of the dynein motor complex, as determined in this study and in our previous report [16], we propose that these interactions are involved in dynein-dependent AR transport (Figure 7). The molecular interactions of REIC/DKK-3 with SGTA and TCTEX-1 link the AR complex and the dynein motor complex and provide new insights for understanding the molecular mechanisms regulating AR signaling and androgen sensitivity.

SGTA was reported to be implicated in various cellular processes and involved in cell cycle progression and transcription and may play a critical role in oncogenesis [34]. With regard to the anti-oncogenic role of REIC/DKK-3, we found that the expression level of TCTEX-1 positively correlated with the incidence of apoptosis in PC3 human prostate cancer cells overexpressing REIC/DKK-3 (data not shown). Thus, the interaction of REIC/DKK-3 with SGTA and TCTEX-1 is implicated in cancer-related signaling suppressed by the tumor suppressor REIC/DKK-3. This finding, together with the fact that TCTEX-1 plays critical roles as an adaptor between the dynein motor complex and various cytoplasmic molecules [17–21], suggests that the interaction of REIC/DKK-3 with SGTA and TCTEX-1 modifies anti-oncogenic signaling at least partially via the dynein motor-mediated protein trafficking system. An important future step will be to clarify the regulatory roles of the cytoplasmic dynein motor complex in carcinogenic and oncogenic processes.

The current study revealed that cytoplasmic REIC/DKK-3 could functionally upregulate dynein motor-dependent AR signaling via its interaction with the co-chaperone SGTA. We consider that the interaction between REIC/DKK-3 and SGTA maintains and activates the physiological AR signaling. If the balance of the REIC/DKK-3-SGTA interaction collapses due to a reduction in REIC/DKK-3 or due to an increase in SGTA expression, the modification of androgen sensitivity may affect the physiological functions of various androgen-sensitive tissues. In addition, the reduced expression of the tumor suppressor REIC/DKK-3 may be of significance for understanding the downregulation of androgen sensitivity during the development of androgen-independent prostate cancer. Recently, the inhibitory roles of SGTA have expanded to include the chaperone-dependent modulation of glucocorticoid, progesterone, and androgen receptor activity [25]. Therefore, the interaction between REIC/DKK-3 and SGTA provides novel insights to improve the understanding of not only their molecular functions but also those of steroid receptor transport and dynein motor axis-dependent signaling.

## MATERIALS AND METHODS

### Yeast two-hybrid assay

Yeast two-hybrid assay with cDNA libraries derived from a normal human heart and prostate and from a prostate adenocarcinoma was conducted using the ProQuest two-hybrid system (Invitrogen, Carlsbad, CA) as described previously [16]. Briefly, human REIC/DKK-3 full-length cDNA was cloned into the SalI and NotI sites of the bait vector pDBLeu. The REIC/DKK-3 expression vector and the human cDNA library that had been cloned into the pPC86 vector (Invitrogen) were co-transfected into *Saccharomyces cerevisiae* strain MaV203. Positive MaV203 clones were selected on appropriate selection plates supplemented with the β-galactosidase substrate. Transformation, plasmid isolation, construct identification and yeast lysate preparation were performed according to the manufacturer's protocols.

### Cell culture

The human embryonic kidney cell line 293T and the human prostate cancer cell lines PC3 and LNCaP were provided by American Type Culture Collection (Rockville, MD). The normal human foreskin fibroblast cell line Hs68 was provided by JCRB Cell Bank (Osaka, Japan). The 293T, LNCaP and Hs68 cells were maintained in Dulbecco's modified Eagle's medium (DMEM) (Wako, Osaka, Japan), and PC3 cells were maintained in Ham's F12 medium (Wako) supplemented with 10% fetal bovine serum (FBS), penicillin (50 IU/ml) and streptomycin (50 μg/ml) under a humidified atmosphere of 5% CO_2_ at 37°C.

### PD assay

We constructed halo-tagged human REIC/DKK-3 in the pFN21A vector (Promega, Madison, WI) and human SGTA in the pF1K vector (Promega). To generate hemagglutinin (HA)-tag fusion proteins, the XhoI/EcoRI fragment of SGTA cDNA was cloned into the pMACS Kk.HA-C vector (Miltenyi Biotec, Bergisch Gladbach, Germany). The expression of Halo- and HA-tagged constructs was induced in 293T cells using FuGENE HD Transfection Reagent (Promega) and PC3 cells using Lipofectamine 2000 Reagent (Invitrogen), and the transfected cells were grown for 48 hours. Then, the cells were harvested by centrifugation and washed with phosphate-buffered saline (PBS). The cells were lysed in Mammalian Lysis Buffer (Promega) with Protease Inhibitor Cocktail for 15 min, and the cellular debris was cleared by centrifugation. In total, 100 ¼l of Magne HaloTag Beads (Promega) equilibrated with TBS containing 0.05% IGEPAL CA-630 (TBS+) was added to the supernatant. The samples were incubated for 20 minutes at RT with rotation. The supernatant was discarded, and the resin was washed three times with TBS+ and suspended in SDS-PAGE loading buffer. The samples were analyzed by WB using anti-Halo antibody (1:1000; G9281, Promega), anti-HA antibody (1:2000; MBL-561, Medical and Biological Laboratories, Aichi, Japan) or anti-FLAG antibody (1:1000; R4-TP1411100, Recenttec, Taipei, Taiwan) and horseradish peroxidase-conjugated anti-rabbit IgG antibody (GE Healthcare, Waukesha, WI). Blots were developed using the EzWestLumi plus reagents (ATTO, Tokyo, Japan). In the experiments performed to interfere with SGTA-SGTA dimerization by the expression of REIC/DKK-3, a construct containing REIC/DKK-3 in the pM vector (Clontech Laboratories, Mountain View, CA) was used to interfere with the binding of Halo- and HA-fused SGTA proteins.

### Mammalian two-hybrid assay

For the mammalian cell two-hybrid assay, various lengths of human REIC/DKK-3 and SGTA cDNA were cloned into the BamHI and MluI sites of the pM GAL4 DNA-binding domain cloning plasmid (GAL4 DBD) and pVP16 transactivation domain cloning plasmid (VP16 AD) (Clontech Laboratories), respectively. The templates for each length of human REIC/DKK-3 and SGTA were generated by PCR amplification using appropriate primer pairs. Approximately 2 × 10^5^ 293T cells on 24-well plates were co-transfected with 100 ng of pM, 100 ng of pVP16, 100 ng of pFR-Luc firefly luciferase reporter plasmid (Promega) and 2 ng of phRL-TK *Renilla* luciferase reporter plasmid (Promega). The cells were harvested at 48 hours after transfection, and luciferase activity was measured using a dual-luciferase reporter assay system. Luciferase activity was normalized to *Renilla* luciferase activity [35]. DBD- and VP16-fused human SGTA constructs were introduced into 293T cells transfected with the REIC/DKK-3 constructs in the pMACS Kk.HA-C vector to examine the interference of SGTA-SGTA dimerization by REIC/DKK-3.

### Immunostaining

Immunocytochemical co-staining for REIC/DKK-3 and SGTA in Hs68 cells was performed using the mouse monoclonal anti-REIC/DKK-3 antibody raised in our laboratory (1:100 dilution) and rabbit polyclonal anti-SGTA antibody (1:100; sc-292025, Santa Cruz Biotechnology, Santa Cruz, CA). Cells were plated and cultured to 50–70% confluence in Lab-Tek chambers (Nalgene, Rochester, NY), and then, the cells were fixed in 4% paraformaldehyde in 100 mM phosphate buffer and blocked with 5% normal goat serum in PBS. After the samples were incubated overnight at 4°C with primary antibodies, they were incubated for one hour at room temperature with Alexa Fluor 488 anti-mouse and Alexa Fluor 594 anti-rabbit secondary antibodies (Invitrogen). To stain the nuclei, the cells were incubated with Hoechst 33342 (Dojindo Laboratories, Kumamoto, Japan) for 15 minutes at room temperature. The fluorescent staining was visualized and analyzed under a fluorescence microscope system equipped with an analytical software program (BZ-9000, Keyence, Osaka, Japan).

### Transactivation assays and AR nuclear translocation analyses

PC3 prostate cancer cells (2 × 10^5^/well in 24-well plates, 500 μL medium/well) were transfected with 100 ng of pEGFP-C1-AR vector containing full-length AR (Plasmid ID: 28235, Addgene, Cambridge, MA) [36], 100 ng of p159-pPr-luc vector containing a firefly luciferase reporter gene downstream of the rat probasin promoter (Plasmid ID: 8392, Addgene) [37], 100 ng of pFN21A-SGTA, 100 ng of pMACS Kk.HA-C-REIC/DKK-3 and 25 ng of phRL-tk *Renilla* luciferase reporter plasmid using Lipofectamine 2000 Reagent. At 48 hours after the cells were transfected, they were treated for 24 hours with vehicle control (ethanol) or DHT (Sigma, St. Louis, MO) and then assayed for their luciferase activity using a Dual-Luciferase Reporter Assay System (Promega). We used charcoal-stripped FBS in the AR signaling experiments, where the basal activity indicates the control medium condition without DHT agent. All transfection mixes were balanced with the appropriate empty vectors in terms of the ratio of the expression vectors and total plasmids.

In the analyses of AR nuclear translocation, the pEGFP-C1-AR vector was transfected into PC3 cells, and AR localization was monitored based on GFP fluorescence. At 24 hours after the cells were incubated with DHT (1 nM) in the treatment groups, they were categorized to 3 groups: the nuclear fluorescence was greater than cytoplasmic fluorescence (N > C), cytoplasmic fluorescence was greater than nuclear (N < C), or even distribution when the fluorescence intensities of the nucleus and cytoplasm were indistinguishable (N = C). Images containing at least 100 cells were evaluated for each treatment, and the cellular category for AR localization was determined [38].

### RT-PCR

Semiquantitative RT-PCR was performed using a commercial kit (Reverse Transcription Kit, Promega) according to the manufacturer's instructions. Parental PC3 and LNCaP cells and pEGFP-C1-AR-transfected PC3 cells under the indicated plasmid co-transfections were treated with 1 nM DHT. After the cells were incubated for 5 days, the mRNA levels of endogenous PSA were examined by RT-PCR. One microgram samples of total RNA obtained from the cells were used for the analyses. The PSA primers for amplifying 217 base pairs (bp) of the human PSA cDNA were 5′-GAGGTCCACACACTGAAGTT-3′ (sense) and 5′-CCTCCTGAAGAATCGATTCCT-3′ (antisense) [39]. In total, 452 bp of GAPDH mRNA was amplified as the internal control.

### Western blotting

Total protein was extracted from the cells, and Western blotting analysis was performed as described previously [40,41]. Approximately 50 μg samples of the extracted protein were analyzed with the following specific primary antibodies: mouse monoclonal anti-REIC/DKK-3 raised in our laboratory, rabbit polyclonal anti-SGTA (sc-292025, Santa Cruz Biotechnology), and anti-β-actin (sc-69879).

### Apoptosis assay

Adenoviral vectors expressing the full-length human REIC/DKK-3 gene (Ad-REIC) and Ad-LacZ were prepared as noted previously [2]. PC3 prostate cancer cells (1 × 10^5^/well) were seeded in six-well plates and incubated for 24 hours. The cells were transfected with pEGFP-N1 (Clontech Laboratories), a TCTEX-1-shRNA plasmid (sc-43319-SH, Santa Cruz Biotechnology) or TCTEX-1 expression plasmid based on pcDNA3.2/V5/GW/D-TOPO (Invitrogen) for six hours, and the medium was exchanged for fresh complete medium. The transfection efficiency of the EGFP plasmid was typically more than 60% at 48 hours after the plasmid treatment. At 24 hours after the cells were transfected with plasmid, they were further treated with Ad-LacZ and Ad-REIC at a multiplicity of infection (MOI) of 50 in serum-free medium for two hours, and then the medium was exchanged for fresh complete medium. After the cells were incubated for 48 hours, Hoechst 33342 stock solution was added to the medium, and the percentage of apoptotic cells was analyzed by fluorescence microscopy as described previously [7].

### Statistical analysis

The data are presented as the means ± SE. An unpaired Student's *t*-test or analysis of variance was performed to analyze the significance of the differences between the two groups. The differences were considered significant for values of p < 0.05.
